# Real-Time, Continuous Monitoring of Tissue Chips as an Emerging Opportunity for Biosensing

**DOI:** 10.3390/s25165153

**Published:** 2025-08-19

**Authors:** John S. Cognetti, Benjamin L. Miller

**Affiliations:** 1 Departments of Biomedical Engineering and Dermatology, University of Rochester, Rochester, NY 14627, USA; cognetti@kth.se; 2 Institute of Optics, University of Rochester, Rochester, NY 14627, USA

**Keywords:** microfluidics, photonics, biosensors, organ-on-a-chip, microphysiological systems, tissue chip

## Abstract

Tissue chips (TCs), otherwise known as organs-on-a-chip (OoC), organ chips (OCs), or microphysiological systems (MPS), are rapidly gaining prominence as an extension of or even replacement for traditional animal models of disease physiology. They also have recognized utility in the context of drug development: for example, data from TCs can now be submitted in place of some animal testing to the FDA. In principle, TCs are structured to allow measurement of any number of outputs that yield information about the tissue. However, to date, measurements made during experiments with TCs have been largely restricted to immunofluorescence microscopy and benchtop assays performed on media extracted from the cell culture within the device. With the development of biosensors that are sensitive and have an ever-shrinking footprint, on-board biosensing is now in the early stages of exploration. This review discusses the importance of tissue chips and the advances in sensing that will aid the complexity and utility of tissue chip research moving forward. We cover several sensing modalities, including electrical and optical sensing modes. Finally, challenges and opportunities for the future are discussed.

## 1. Introduction

Traditional in vitro cell culture, where cells are isolated and then proliferated or maintained on an artificial substrate, has been practiced since the mid-1900s. This developed into high-volume or high-throughput culture of cells in flasks or well plates, allowing for the study of cells on a large scale. The complexity and biological relevance of traditional cell culture is limited, however, as monotypic cell culture cannot replicate the complexity of an organ or organism.

In the last twenty years, microtechnology developed in the semiconductor industry has been realized as an important tool for controlling cell culture via the production of microfluidic devices. Microfluidics involves the use of microscale channels to deliver small fluid volumes to small cell-culture areas, greatly increasing efficiency [1,2,3,4]. As microfabrication techniques became more sophisticated, so did microfluidic devices, enabling simultaneous culture of multiple cell types and delivering mimics of organ-level physiology [1]. These microfluidic cell-culture platforms are now variously known as microphysiological systems (MPS), tissue chips (TC), organ chips (OC), organ-on-a-chip devices, or organs-on-chips (OoC). For simplicity, we will use TC for the remainder of this review, with the understanding that this encompasses MPS and OC/OoC systems. These devices mostly exist as transparent or semi-transparent glass or polymer-based devices, with the term “chip” remaining as a relic of their origin in semiconductor manufacturing processes.

**Figure 1:**
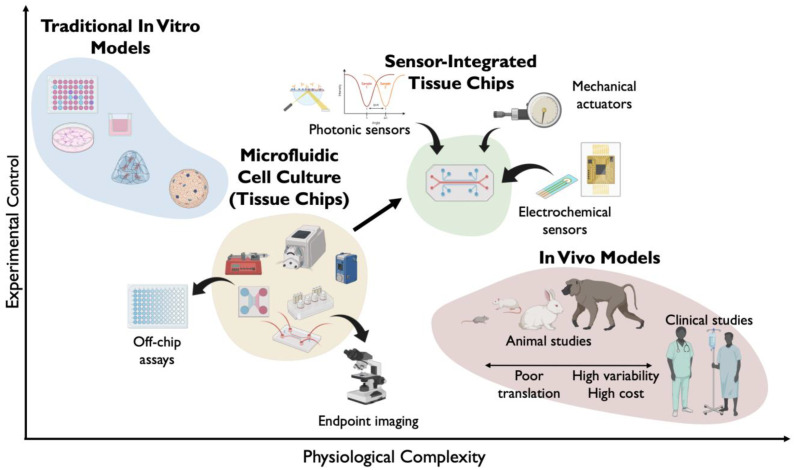
The role of tissue chips (TCs) in modeling complex biology. (Inspired by Ma 2021, Ref [5].) Conventional 2D cell-culture techniques allow high degrees of control, accuracy, and throughput, but are not complex enough to mimic human biology. In vitro models add complexity in the form of 3D cultures, sometimes including matrices or scaffolds, or developmentally accurate organoids. This reduces control and throughput, however. Animals are appropriately complex but introduce vast genetic differences that often do not translate to humans. Higher-order animals and human clinical studies are expensive, and in the case of clinical studies, have unavoidable genetic variability within the large populations necessary for clinical trials, resulting in very small effects at the population level. By incorporating microarchitecture and physiological flow conditions, tissue chips seek to take advantage of the high level of experimental control of traditional cell culture and approach the physiological relevance of animal models while improving translatability by using human cells. Additionally, by incorporating sensors and actuators into TCs, new levels of control and physiological relevance can be reached, while providing high-dimensional data that will improve translation.

While less complex than organs in vivo, the complicated microarchitectures possible with TCs allow researchers to both manipulate the cells within a TC and to measure their responses [5]. This includes maintaining physiological flow characteristics, adding extracellular matrix (ECM) components to the substrate (3D ECM scaffolds), adding membranes to support tissue barriers in multichannel fluidic devices, adding channels for vasculature, and incorporating 3D cultures of multiple cell types called spheroids or organoids. As a result, TCs have the potential to reduce reliance on animal models. The relationship of TCs and sensor-integrated TCs to traditional in vitro methods and animal models is highlighted in Figure 1.

In addition to recreating complex physiology in vitro, TCs allow researchers to observe tissue and measure its behavior in more sophisticated ways than is possible with traditional 2D static cultures. Cells constantly produce biomolecules and release them into their environment. These biomarkers can be used to learn more about the mechanisms behind disease progression and may also present new therapeutic targets. Likewise, the way damaged or diseased cells respond to candidate drugs can be assessed by measuring secreted analytes from cell culture. Standard in vitro techniques for studying how cells behave in response to injury, disease states, or drug treatment include sampling the large volumes of media in which the cells are cultured and measuring parameters such as oxygen content, pH, and quantities of metabolites or protein secretions using benchtop assays, such as the enzyme-linked immunosorbent assay (ELISA). These processes are laborious and are conducted post facto on media sampled from the TC. As such, temporally and spatially resolved information about the production of analytes of interest is lost.

Recognition of the need for continuous monitoring of TCs is driving rapid advances in the development of biosensors for TC integration. Beyond the development of academic models, many companies have been founded to address this need. Since Emulate was founded out of the Wyss Institute at Harvard in 2013, there have been dozens of startups attempting to build better TC models, and this includes the incorporation of on-board sensors for real-time monitoring. For example, Mimetas (Leiden, The Netherlands) makes TCs with embedded TEER sensors, and Hesperos (Orlando, FL, USA) produces chips with embedded microelectrodes for measuring electroactive cellular activity. Some companies, like TissUse (Berlin, Germany), Nortis (Woodinville, WA, USA), and CN Bio (Cambridge, UK), market their chips as being compatible with TEER or external sensor probes, but without truly embedded sensors. Thus, there remains an opportunity in the market for real-time sensing in TC models that would be useful to academic groups and pharmaceutical companies alike.

**Figure 2:**
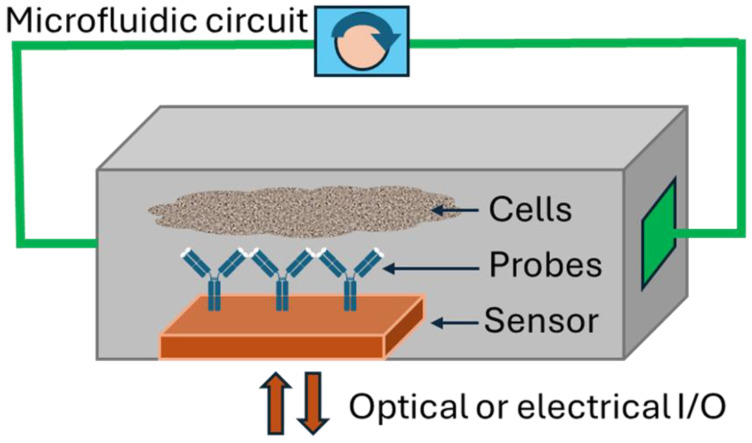
Simple general schematic of sensor integration with TCs. Incorporation of a sensor (or many sensors, potentially operating in different modes) into the microfluidic system close to cells under study allows for continuous analysis of the TC. Here, “cells” may be several different cell types, arranged in a 3D matrix designed to mimic the organization of an organ.

The use of physical sensors and off-chip assays in TCs has been reviewed elsewhere [6,7,8]. This review will therefore focus on the motivations behind advancing sensor-integrated TCs and look at the ways incorporation of novel biosensors provides access to previously unobtainable data, changing the way we study biology and find new therapeutics for diseases. Shown schematically in Figure 2, the general goal is to place continuously operating sensing elements within the microfluidic flow path, and as close to the cells as possible, to take advantage of high local concentrations and reduce latency. While we cannot be comprehensive, and we note that this field is rapidly evolving, we have endeavored to cover key studies in the literature through early 2025.

## 2. Motivation for Development of TCs

### 2.1. Limitations of Animal Studies

To date, animal studies have been considered the gold standard for addressing basic physiological questions as well as assessing the safety and efficacy of new drugs. The mechanisms behind complex physiological processes and drug responses are a function of tissues, organs, and even multi-organ levels of complexity. Thus, living organisms have been sought as the ideal model for replicating the conditions under study. Additionally, animals are mandatory for toxicity or experimental disease studies that would be unethical to perform in humans. That is why animal use in biomedical research has been predominant over the past century, with current estimates of over 100 million animals being used in the 2017–2018 reporting year, mostly consisting of mice and rats [9].

Despite these advantages, animal research has many limitations. While the physiology of animals is similarly complex to that of humans, differences between the physiology of humans and lower mammals are well known [10,11,12]. For example, small differences in the peptide sequences between human and analogous animal target proteins can result in differences in drug efficacy. Differences between the biology of animal disease models and that of humans are believed to be significant contributors to the staggering 90 percent of successful preclinical animal trials failing to proceed to the market [13,14,15]. Higher-order mammals are better models for humans, but their use is ethically troublesome and extremely costly.

### 2.2. Limitations of Clinical Studies

Human clinical studies are often used to measure differences in disease progression or drug responsiveness in individuals with varying genetic and environmental profiles to help understand disease mechanisms and thus reveal or help validate therapeutic targets. Massive numbers of patients are required to make statistically significant conclusions about disease pathways. For example, the Phase III clinical trial for the Pfizer/BioNTech mRNA vaccine for COVID-19 used over 43,000 subjects [16]. While some diseases consist of a single mutation (e.g., spinal muscular atrophy, SMA) and can be targeted pharmacologically with relative ease, multifactorial diseases, such as Alzheimer’s, require vast numbers to identify new treatment targets. The costs associated with such high-volume studies quickly become prohibitive. The vast heterogeneity in human populations due to both genetic and environmental factors may complicate the understanding of clinical study results and make it difficult to isolate mechanisms of diseases and drug action.

### 2.3. Applications of TCs: Disease Modeling, Drug Development, Human Development, and iPSC-Based Analysis

**Figure 3:**
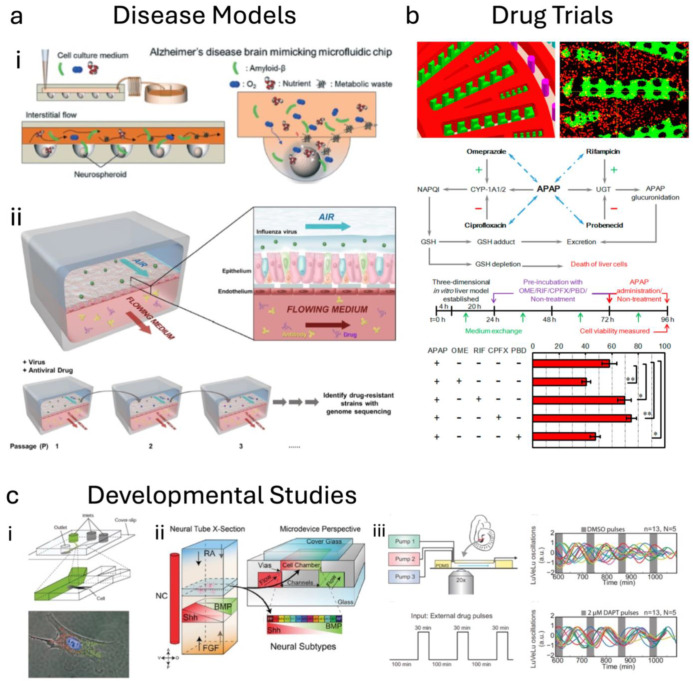
Tissue chip applications. (**a**) Tissue chips have been used to model many diseases, including long-term degenerative diseases like (i) Alzheimer’s (reprinted from Park 2015, Ref. [24] © Royal Society of Chemistry), or short-term infectious diseases like (ii) influenza (reprinted from Si 2021, Ref. [41] © American Society for Microbiology). (**b**) A study used a liver chip model to look at interactions between many drugs; * and ** indicate levels of statistical significance (reprinted from Ma 2016, Ref. [43] © Royal Society of Chemistry). (**c**) Microfluidic organ chips can be used to study development by (i) applying chemical gradients to the same cell (reprinted from Takayama 2001, Ref. [50] © Springer Nature), or (ii) tissue (reprinted from Demers 2016, Ref. [51] © The Company of Biologists). Additionally, (iii) multi-pump devices can be used to flow analytes cyclically and study their interactions (reprinted from Sonnen 2018, Ref. [52] © Cell Press).

Due to the lack of translation from animal studies and poor outcomes in heterogeneous populations following successful clinical trials, TCs have begun to be used to study the underlying pathophysiological processes in diseases and the effect of candidate therapeutics on these properties (Figure 3). TCs provide added complexity to more accurately mimic in vitro cellular environments, as well as reconstruct the pathologies seen in diseases through genetic manipulation or external stimulation.

As discussed briefly in the introduction, the development of lithographic techniques, originally for the semiconductor industry, has enabled the advancement of TCs through micropatterning of cell-culture substrates. By geometrically controlling where cells are deposited within the device, cells or even organelle [17,18,19] types may be studied in a complex biological environment. While other technologies (discussed later), including laser cutting and 3D printing, are undergoing rapid growth in the field, microfluidic prototyping has been particularly enabled by the patterning of elastomeric material (e.g., polydimethylsiloxane, PDMS) in a process called “soft lithography”. First described by the Whitesides group in 1998 [20], the process involves the photolithographic patterning of microscale structures on silicon wafers, which are then used as a mold for the casting of PDMS. When the PDMS cures, it can be removed from the wafer with the photoresist pattern now embedded in the PDMS. This technique has been widely used in the TC field for controlling the precise size and location of microfluidic channels, but also to introduce features into the channels to further control flow patterns or to control the location of particular cell types or their orientations [21]. Diseases such as Alzheimer’s [22,23,24,25], fatty liver disease [26,27,28], and muscular dystrophy [29,30,31] have all been modeled in microfluidic TC systems by controlling cell architecture in elastomer-based devices. Bacterial and viral infections have also been studied using similar TC models, such as hepatitis in a liver chip [32,33], pseudorabies kidney infection [34], and influenza infection in a lung chip [35,36]. Multi-organ TCs, produced by linking more than one single-organ TC microfluidically, can be used to study more complex pathologies or interactions between pathologies. For example, cancer metastasis involves crossover between multiple tissue types, making it an ideal application for linked TCs, with a multi-organ lung cancer metastasis model already published [37].

The potential for TCs to aid in the discovery and development of new drugs has long been recognized due to their small size and scalability for medium- to high-throughput testing with human cells. TCs seek to obtain a suitable level of complexity to mimic animal studies, but with human cells to mitigate some of the translation issues mentioned earlier. The concept of a “body-on-a-chip”, allowing for multiple TCs to respond to a drug and interact with each other, is already being explored [38,39].

As an example of validating TCs for drug evaluation, Khalid et al. assessed the efficacy of cancer drugs against lung cancer cells [40], measuring pH and cell viability changes using built-in electrical sensors. More recently, the Ingber group has used lung chips to identify potential therapeutics for COVID-19 [41]. High-throughput platforms are being explored as well, interfacing with common lab interfaces such as 96-well plates [42] to screen drugs more efficiently [43] and attenuate the high costs associated with pharmaceutical development. This need has been recognized by many world governments [44], and in 2022, the US Congress passed the FDA Modernization Act 2.0, which allows for TC data in preclinical drug trials [45]. We already have examples of this being implemented with Azeliragon [46], the first FDA-approved clinical trial built in part on preclinical data from TCs [47]. As mentioned at the outset, recent press releases from both the FDA and NIH indicate an even stronger emphasis on replacing animal models with TCs in the US [48,49].

TCs can also be used to measure the efficacy of delivery methods. Here, incorporation of endothelial barriers is critical, since drugs must pass through the vascular endothelium to reach target tissues. Drug developers are attempting to target drugs toward particular tissues and need ways to rapidly determine relative uptake in multiple tissue types in humans. Thus, TCs and multi-organ TCs are critical for determining these properties of drugs. A study from the Vunjak–Novakovic group looked at just this, measuring the presence of drugs in various tissue compartments within a single device, and investigated the effect of including an endothelial barrier between the cells and the source of the drug (representing the bloodstream) [38].

A special case of endothelial barrier study applicable to drug development is the blood–brain barrier (BBB). The BBB is a selective endothelial barrier present only in the endothelium of brain capillaries and serves to protect the brain from injury and infection. Tight junctions (TJs) consist of both physical paracellular diffusion barriers (in the form of zipper-like proteins on the membranes of brain endothelial cells) and efflux transporter proteins that actively expel hydrophobic molecules that have entered the cell via passive diffusion or endocytosis. TJs are important for preventing local inflammation in the brain during injury and for preventing pathogens from entering the brain. However, they also inhibit drugs (which are often hydrophobic) from diffusing into the brain, making treatment for brain diseases difficult.

TCs have been used to study drug passage across the BBB to help identify therapeutics that will be effective in humans. The Shuler group, using a brain endothelial cell and astrocyte coculture model under physiological flow conditions, measured permeability of the barrier to doxorubicin and other small molecule drugs, while monitoring transendothelial electrical resistance [53] (TEER), a surrogate for barrier integrity (discussed later). There are many additional physiological parameters that define drug response in tissue, though, so adding sensor modules to tissue chips to better define how cells are responding to drugs could increase the value of tissue chips used in drug studies.

TCs can also be used effectively in the study of human development [54], including the influence of fluid flow on the development of intestinal epithelium [55], breathing forces on lung epithelium [56], and the effect of inclusion of endothelium on lung inflammatory responses [57]. Additionally, microfluidics has been used to create artificial biochemical gradients and study their effects on single cells [50] or neural tube tissue [51], while multi-pump TCs have been used to study the effect of oscillating patterns of various signaling molecules in developmental pathways [52].

In 2006, Takahaski and Yamanaka published a protocol for reverting differentiated tissue from mice into pluripotent stem cells (induced pluripotent stem cells, or iPSCs) [58]. A protocol suitable for human iPSCs followed in 2007 [59]. Using the somatic cells from any individual (initially dermal fibroblasts), treatment of the cells with a combination of transcription factors reverts them into a pluripotent state. In the years since that pioneering discovery, researchers have developed simpler protocols using more accessible cell types, such as keratinocytes from a single hair pluck [60].

Once pluripotent cells are verified as such, they can then be differentiated into any cell type. The late 2000s/early 2010s saw the development of many differentiation protocols, and a wide variety are currently publicly available. Many were adapted from previously discovered work in embryonic stem cells (ESCs). As an example, differentiation protocols developed in ESCs for motor neurons [61] were then adapted to human iPSCs from an individual with amytrophic lateral sclerosis [62] (ALS), producing neurons and glia with an ALS genotype. These protocols have since been turned into a viable sporadic ALS model with proteinopathy and may be used in drug efficacy studies [63].

The implications of iPSCs are significant. In principle, cells from any individual can be used to generate an in vitro model of aspects of that individual, or “TC Twin”. This includes adults who have developed certain diseases or simply individuals of a certain genotype of interest. Therefore, disease progression and pathophysiology can be studied in specified genotypes, helping direct individual treatment plans clinically. Given that drug response varies drastically in a heterogeneous population, as already discussed, iPSC-based models can be used to determine a drug’s efficacy and toxicity among individual patients to further optimize treatments for diseases. When integrated into TCs, such efforts may be viewed as “clinical trials on a chip” [45].

Human iPSCs had already been used to screen drugs in vitro (such as drugs to reduce Aβ production in neurons [64]); however, this is usually done in well plates requiring large quantities of cells and reagents, quickly becoming prohibitively expensive. TC models reduce the costs associated with iPSC use, given their lower cell and reagent burden. TCs have begun incorporating patient-derived iPSCs to study drug response [65], disease pathophysiology [66], and developmental biology [67]. Some models even incorporate multiple organ modules with an endothelial barrier to simulate in vivo drug kinetics [38], as endothelial cells play a large role in drug access to tissues [68,69].

## 3. Incorporation of Physical and Chemical Sensing Modalities into Tissue Chips

As readers of this journal are well aware, sensors can be split into many categories according to what they are sensing or the way they transduce signals into usable forms. Physical sensors monitor physical parameters such as temperature or the barrier permeability (i.e., TEER) of the system. Chemical sensors of utility in TCs include pH and oxygen concentration, often measured with electrical sensors. Biosensors measure the presence of specific bioanalytes in solution, often using capture probes such as antibodies or aptamers, but may also identify specific analytes by spectral fingerprints, such as with Raman scattering-based sensing or sensing of redox-responsive analytes via voltammetry. In the following sections, we provide an overview of the various parameters that have been measured in tissue chip devices, what sensor characteristics are most important to cell-based studies, and what advantages and disadvantages they each have for TC integration.

### 3.1. Oxygen Sensors

For in vitro tissue models to sustain proper metabolic and expression patterns, they must maintain physiological oxygen concentrations within them. Either an excess [70] or deficiency [71] in molecular oxygen will result in cell damage or death, and each tissue type has a unique oxygenation profile [72]. The various materials used in making microfluidic devices have different permeabilities to oxygen gas [73], highlighting the need for continuous monitoring of oxygen content in tissue chips.

**Figure 4:**
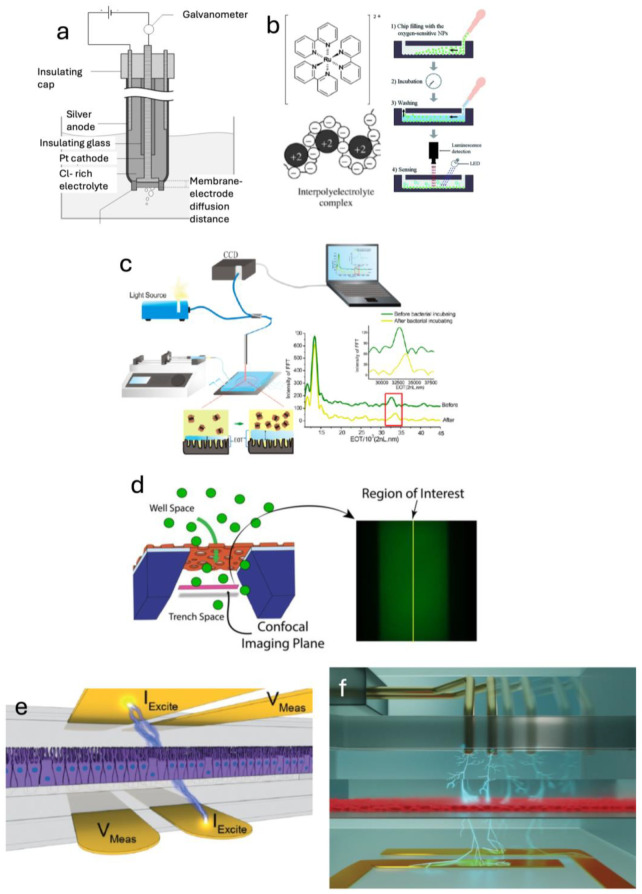
Physical and chemical sensors. (**a**) A Clark electrode, which relies on electrochemistry to measure dissolved oxygen content (Reprinted from Clark 1953, Ref. [74] © American Physiological Society). (**b**) Oxygen-sensitive dyes (left—reprinted from Chang-Yen 2002, Ref. [76] © Elsevier) which can be incorporated into microfluidic devices (right—reprinted from Lasave 2015, Ref. [79] © Royal Society of Chemistry Publishing). (**c**) The pH in a microfluidic device can be determined by measuring thickness changes in a pH-sensitive polymer layer (Reprinted from Tang 2013, Ref. [81] © American Chemical Society). (**d**) Permeability of a barrier to fluorescent particles can be measured with a confocal microscope in a region of interest (Reprinted from McCloskey 2022, Ref. [82] © John Wiley & Sons). (**e**) TEER electrodes incorporated into a microfluidic organ chip device (Reprinted from Henry 2017, Ref. [83] © Royal Society of Chemistry). (**f**) A movable electrode is used for spatially-resolved TEER measurements (Reprinted from Renous 2021, Ref. [84] © Royal Society of Chemistry).

Researchers have incorporated oxygen sensors into TCs to monitor oxygen levels, either to ensure cell health or to carefully control oxygen concentrations in studies of hypoxia. Oxygen sensors may be either electrochemical, optical, or colorimetric in nature. Electrochemical oxygen sensors operate by measuring current in response to the reduction of oxygen close to a pair of electrodes, which is proportional to the concentration of oxygen. This was first demonstrated by Clark et al. in 1953 in blood [74] (Figure 4a) and has been miniaturized since [75], allowing for incorporation into a tissue chip device. Standard lithography techniques allow for the precise deposition of electrodes within the confines of a microfluidic channel, making electrochemical methods appealing for tissue chip sensing.

Optical methods for quantifying oxygen involve the quenching of fluorescence by oxygen. Luminophores using ruthenium have been most successful, detecting oxygen in microfluidic settings [76] (Figure 4b). Additionally, dyes encapsulated in polymer gels [77,78] or nanoparticles [79,80] have been successful in measuring oxygen content in microfluidic systems.

Recently, visible light-emitting nanoparticles using derivatives of earlier oxygen-sensitive nanoparticles have been used to obtain colorimetric readouts of oxygen concentration [85]. This rapid, visual readout (albeit semiquantitative) is particularly useful as a monitor for tissue chips required to maintain physiological oxygen levels. The use of nanoparticle-based oxygen sensors additionally allows for versatile microfluidic incorporation, making oxygen sensors in TCs a standard inclusion (reviewed extensively elsewhere [86]).

The methods for fabricating optical and colorimetric oxygen sensors consist of the synthesis of the active materials coupled with liquid-phase deposition of the material within a microfluidic device. This often means manual pipetting and manual alignment of device layers, but microarrayers or versatile lithographic techniques may make these methods scalable in the near future. A recent study by the Loskill group used a commercial microdispensing device to deposit liquid-phase platinum-based oxygen-sensitive dye in a microfluidic device used to study pancreatic metabolism, demonstrating the promise of these methods [87].

### 3.2. pH Sensors

In addition to dissolved oxygen content, the pH of a TC is critical for proper expression profiles and the overall health of cells. The pH of blood is tightly regulated within the range of 7.35–7.45. Standard cell-culture protocols take measures to ensure stable pH, as both high and low pH have been shown to negatively impact cells [88], as H^+^ ions (protons) can protonate or induce post-translational modifications in enzymes or receptors and therefore alter cell homeostasis [89,90]. Cell-culture media is often supplemented with a zwitterionic buffer, such as HEPES, to resist changes in pH in either direction. In concert with this, incubators are maintained at 5% CO_2_, as the dissolved CO_2_ reacts with water to form carbonic acid, which in turn balances the carbonate ions (present in most cell-culture media, as it is necessary for normal metabolic function in cells), to maintain the pH at physiological levels.

Although most standard cell-culture protocols do a good job of maintaining a physiological pH, it is useful for TCs to monitor pH continually, especially where nonstandard pH is required. For example, cancer cells function optimally at a lower pH [91,92]. Additionally, the pH of the interstitial space in various tissues is not well understood [93], and the pH of the interstitial space will depend on the metabolic characteristics of the cell types present, secreted proteins or neurotransmitters, or the expression of certain enzymes. Therefore, pH sensor-integrated tissue chips could help understand the role pH plays across different tissues.

Current pH sensors used in TCs work through a variety of modalities. Electrochemical pH sensing (which simply measures the potential difference between a working and reference electrode in an electrolyte solution) has been used [94,95,96], but optical methods, such as measuring absorption ratios of indicator dyes [40,97,98] (e.g., phenol red), are becoming popular. Thin films may be used to determine pH, either by mesoporous containment of pH-sensitive dyes [99] or by measuring the pH-dependent swelling of chitosan-based films via reflectometry [81].

In the context of a TC, the fabrication method, form factor, and data acquisition modality are all important for determining which type of sensor will work best. Optical measurements of absorption in the media do not require any materials to be deposited in the fabrication process, but they necessitate expensive and bulky optical equipment to be placed in immediate proximity to the chip. Electrodes require precise and expensive fabrication, but wires carrying the signal can be easily routed for a more convenient experimental setup. While the optimal sensor type depends on the nature of the overall chip setup, the choice will ultimately come down to cost, which includes both the cost of fabrication and the cost of external measurement equipment.

### 3.3. Fluorescence for Monitoring Barrier Permeability

In addition to chemical sensing of various physiological parameters, it is possible to visually track particles in a TC using fluorescent dyes. This is particularly important for understanding flow patterns, or a membrane or tissue barrier’s permeability to analytes of varying sizes.

For example, the permeability of a cellular barrier, such as an epithelial monolayer, can be determined by quantifying the number of fluorescent molecules that are able to pass through it. Permeability can be calculated by recording the relative fluorescence intensity on the side of the barrier opposite the channel where the fluorescent molecule is introduced. This has been done with artificial membranes [82] and tissue barriers in a tissue chip model [100,101].

In a device where the fluidic channels are stacked on top of each other vertically, traditional microscopy captures the light from both channels. However, a confocal microscope can be used to isolate fluorescence from a specific channel and calculate the concentration of fluorophore in that channel from the fluorescence intensity in a given plane, establishing a permeability value for the barrier [82]. Additionally, dyes of different molecular weights (including high molecular weight fluorescent derivatives of dextran) can be used to determine size restrictions on barrier permeability.

### 3.4. TEER

Tissue barriers are an important subject of many TC studies. For example, the blood–brain barrier [102,103,104,105], intestinal epithelial barrier [55,106,107], and skin [108,109,110] have all been studied in TCs. The integrity of such a barrier is essential for understanding cellular response to external stimuli or disease states, as dysfunction in these barriers can indirectly report on gene expression or cell health [25,111,112,113].

Characterizing tissue barriers in vitro via microscopy has been troublesome, as the materials used in microfluidic channels may scatter light. Thus, a nonvisual method for determining barrier integrity, called transepithelial/endothelial electrical resistance (TEER), is often used to track barrier properties, in addition to, or in replacement of, fluorescent imaging or dye tracking, mentioned earlier.

Cell-culture media contains high salt concentrations, and the ions in the solution make the media electrically susceptible. When a voltage is applied across a cellular barrier, ions move across wherever they are free to move through the liquid medium, and the resulting current can be measured by electrodes situated on either side of the barrier. Thus, where the barrier between cells is tight, water cannot permeate between the cells, and ions will not flow, resulting in a low current/high resistance value. TEER, therefore, serves as a surrogate marker of the tightness of the tissue barrier.

In tissue models where the expression of tight junctions is relevant, TEER can be used to track the formation of tight junctions over time. Barrier cells often take days to expand into a full monolayer, and then days more to form tight junctions between the cells. Thus, continuous TEER measurements are useful for informing the observer when the barrier is completely formed, and a barrier-dependent experiment can be started.

Additionally, change in barrier integrity is an important parameter in studies of drug, pathogen, and toxin interactions with tissue, as well as the progression of disease states. TCs have used TEER to characterize barrier function in many such disease models and drug studies. For example, the Ingber group incorporated thin gold electrodes into a two-channel microfluidic device to study the effects of chemicals on barrier disruption in both lung and intestinal epithelia [83]. Since that initial report, this has been done by numerous groups with various adaptations [114,115,116], including high-throughput models [42,117].

However, TEER provides limited information about what is actually happening with a tissue barrier. Current will readily flow through any low-resistance areas anywhere on the area along the cell layer. Since TEER measurements represent an average of the entire volume sampled by the electrodes, any gaps in the barrier will greatly reduce TEER measurements, even if they are very small. TEER measurements become less meaningful for any tissue barrier that is not a perfect, continuous monolayer. Some groups have attempted to get around this issue by designing spatially-resolved TEER, where either an array of electrodes [118] or a single movable electrode [84] can be used. This yields more accurate information, showing high resistance values across most of the barrier and low resistance over holes.

With regard to the design of TCs, TEER can be difficult to implement. Electrodes are often made of metal, making imaging through them difficult. Some groups have evaded this issue by using semiconductor electrodes, such as indium–tin–oxide [40] (ITO), which is transparent. Even so, the resulting wire connections complicate device architecture and require additional off-chip organization to allow for proper integration with analysis instrumentation. This has been done commercially, with Mimetas’ OrganoTEER^®^ module, for use with the OrganoPlate^®^ tissue chip platform, taking TEER measurements from up to 64 chips (arrayed on the OrganoPlate^®^) simultaneously in under a minute. This platform has been used to characterize intestinal epithelial chips in a high-throughput manner [119]. The OrganoTEER^®^ module uses electrodes inserted through the inlet and outlet channels, however, and it should be noted that TEER measurements are sensitive to device geometry and electrode placement [120,121,122], and though self-consistent, a high-throughput TEER module with electrodes inserted into inlet/outlet ports like the OrganoTEER^®^ might not be as accurate as for integrated electrodes.

The use of TEER in TCs is a helpful approximation of barrier functionality, but new models are necessary that will give more direct measurements of barrier integrity, account for minor defects that are spatially confined, and sense specific molecules being produced by the cells making up the barrier.

## 4. Incorporation of Bioanalyte Sensors into Tissue Chip Models

The physical and chemical sensing modalities mentioned above are important for understanding the physiological parameters of a TC and optimizing the data. However, the vast majority of tests for biomolecules in TCs have relied either on immunofluorescence microscopy (which most often requires “killing” the chip) or sampling media and running benchtop assays. On-board biosensing of specific analytes is a widely recognized need, with techniques enabling continuous monitoring of particular interest. A list of sensor-enabled tissue chips is shown in Table 1, including those with biosensors.

One of the earliest TCs to incorporate on-board biosensing was reported by Zhang et al., in which antibody-functionalized electrochemical sensors were used to detect analytes secreted from organoids [39]. Additional optical sensors (photodiodes positioned opposite LEDs of specific wavelengths) measured other parameters: oxygen content (by measuring absorption for an oxygen-sensitive ruthenium-based dye) and pH (by measuring absorbance from pH-sensitive phenol red in media). This represented tremendous progress in on-board sensor incorporation, particularly given the multiparametric data resulting from both optical and electrochemical sensors. Another model from Ortega et al. sensed secreted proteins from a skeletal muscle chip by incorporating commercial biosensor electrodes microfluidically in line with cells, where the cells under study are stimulated by both chemical analytes delivered via microfluidic flow and electric fields induced by actuator electrodes [123]. As with the Zhang device, this is very important for measuring the time course of cell secretion by avoiding sampling of media or using endpoint assays.

While technically exciting, there are several limitations to these models. First, since the separate modules are connected by lengths of tubing, analytes secreted from cells (as well as the drugs used to stimulate the cells) can be lost to adsorption/absorption along the length of the fluidic circuit, resulting in inaccurate inputs and outputs. These designs also place limits on temporal precision, as the analytes of interest are shuttled through long lengths of tubing. One solution to issues with analyte loss would be to bring sensors into close proximity with the cells under study, so secreted analytes are actually closer to the sensors than any absorbent housing material.

### 4.1. Sensitivity Requirements

**Figure 5:**
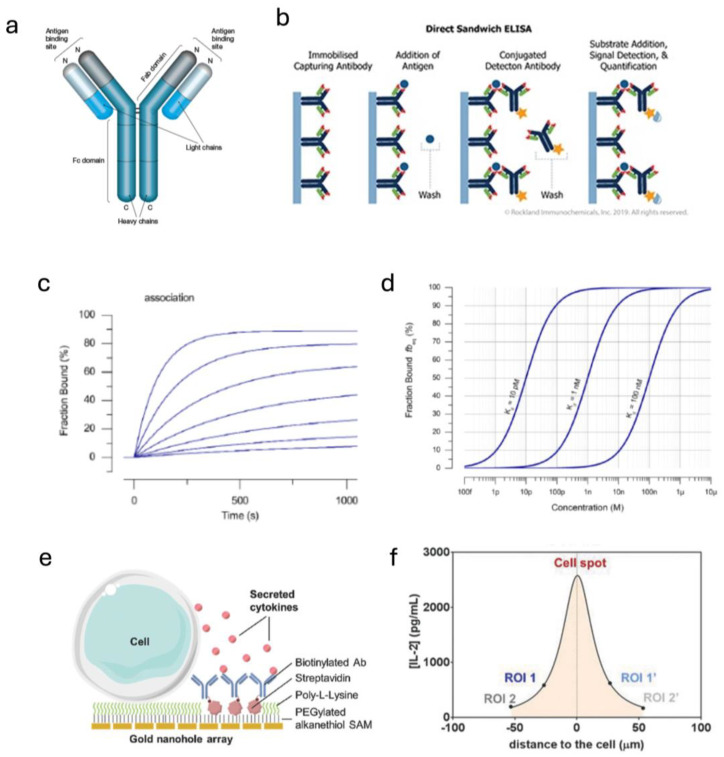
Antibody-based sensors. (**a**) An antibody consists of a species-specific F_c_ region, with two antigen-binding arms, called the F_ab_ regions (ScienceDirect.com). (**b**) An ELISA utilizes antibodies in a multi-step assay. A label-free assay behaves as the second panel, where an antigen binds an immobilized antibody. Labeled or “sandwich” assays then add a fluorescently tagged detection antibody (third panel) or an enzyme-conjugated detection antibody followed by the enzyme’s substrate for additional amplification (PerkinElmer.com). (**c**) A kinetic binding curve showing the bound fraction of analyte over time for increasing concentrations. (**d**) A Langmuir isotherm, showing fraction bound as a function of concentration. Increasing K_d_ moves the curve to the right (dynamic-biosensors.com). (**e**) Sensing cellular secretions in close proximity to the cell yields (**f**) a Gaussian distribution, with higher concentrations closer to the cell (Reprinted from Li 2018, Ref [132] © John Wiley & Sons).

The performance of novel biosensors is constantly improving, with sensitivities down to single pg/mL levels now possible in some cases. This is undoubtedly beneficial for achieving high-quality diagnostic tests, but what sensitivity is actually required for the sensing of biologically relevant concentrations of secreted analytes in a tissue chip? The concentrations of disease biomarkers in blood are sometimes very low (e.g., IL-1β [124,125] and IL-6 [125,126] in Alzheimer’s, and S100B [127] or UCH-L1 [128] in traumatic brain injury), making them difficult to detect. Tests for these analytes often use established clinical laboratory methods, such as enzyme-linked immunosorbent assays (ELISAs), which are time-consuming and costly due to heavy reagent and consumable lab material requirements. However, the labeled nature of ELISAs makes them very sensitive (LODs usually in the 1–100 pg/mL for commercially available sets, e.g., R&D Systems Human IL-1β DuoSet ELISA kit, Catalog # DY201, which has a lower limit of 3.9 pg/mL). A comparison of labeled vs. unlabeled antibody-based assays is shown in Figure 5a,b. Further sensitivity has been obtained by combining these labeled assays with optical or electrochemical sensors, such as photonic ring resonators [129], discussed in detail later.

However, the fundamental limit of sensitivity is determined by the analyte–probe interactions. For example, for a given antibody–antigen pair, the dynamic range is determined by the pair’s affinity (K_d_), with the equilibrium binding relationship given by the Langmuir isotherm binding equation (Figure 5d). This equation describes the percentage of probes on a surface that will be bound as a function of concentration, yielding a sigmoidal curve. Evident from this, there will be a certain threshold concentration below which there will be no analyte bound to the probes, making sensing impossible, and therefore limiting the innate sensitivity of a system using affinity-based probes such as antibodies.

While disease biomarkers in blood, urine, or saliva may be present only in very low concentrations, their concentrations in the tissue from which they originate are actually much higher. The blood concentration consists of only the small fraction that diffuses to nearby blood vessels and then passes through the endothelial barrier into the bloodstream. From in vivo clinical studies, it is known that biomarker concentrations in cerebrospinal fluid (CSF) in neurodegenerative diseases are actually much higher than those found in the blood [130,131]. For diseases in organs not readily accessed by surpluses of fluid, the concentration of important analytes is poorly studied, though it stands to reason that the concentration is actually much higher in close proximity to the cells producing them.

The increased concentration of biomarkers close to their tissue of origin has been corroborated in vitro. Li et al. used optical sensors in a microfluidic system to isolate single cells and measure cytokines secreted from them [132]. Using an array of sensors spatially oriented in varying distances from the cells under study, they found that their sensors measured higher concentrations of secreted analytes in regions of interest closer to the cells. They could then calculate a Gaussian distribution of analyte concentration around the cell, finding higher levels (single ng/mL) at the surface of the cell, even though their sensors were measuring lower levels on the order of 30–100 μm away (Figure 5e,f).

In a TC, many cells are close together, and if presented with the same stimulus, they will secrete the same analytes. Presumably, the high number of cells in a TC (relative to the single-cell study mentioned above) will result in high concentrations of secreted analytes. These observations suggest that sensors brought in close physical proximity to the cells under study do not need the restrictively high sensitivities required for blood diagnostic applications.

### 4.2. Luminescent Sensing of Biomolecules

Fluorescently-tagged antibodies may be injected into the system to measure the presence of specific analytes secreted by cells. However, since the analytes are not fixed in place, an immobilization procedure must occur off-chip so that unbound antibodies can be removed for an accurate measurement. While several studies have used these types of methods to measure proteins produced in cellular systems [132,133], the complexity of the approach and inability to obtain real-time information highlight the need for label-free analyte detection in TCs that do not require additional procedures off-chip.

### 4.3. Electrochemical Sensing of Biomolecules

Many diagnostic applications use electrochemical platforms for measuring the presence of specific analytes near a sensor surface. Electrochemical sensors can sense analytes near functionalized electrodes through a number of modalities. Primarily, small molecule analytes, such as lactate, can be sensed because of either inorganic metal oxide surfaces [134] or enzyme-functionalized electrodes [135] that oxidize lactate, releasing electrons that contribute to the current in the electrodes. In the case of larger molecules, such as proteins or nucleic acids, the electrical properties of the substrate, particularly its resistivity, are affected by the binding of analytes to probe molecules functionalized on the surface. Thus, measured electric potential or current values can be used to calculate the concentration of analytes in solution in close proximity to the electrodes.

In a typical three-electrode sensor, a working electrode consists of a conductive metal (e.g., gold, platinum, graphene) functionalized with the oxidation catalyst, antibody, or other probe. Nearby, there is a counter electrode for receiving the current generated at the working electrode. A reference electrode (usually silver–silver chloride, Ag/AgCl, though this can leach off cytotoxic silver ions [136,137]) is used to measure or set the potential, and it is usually spatially separated from the working electrode so that the potential may be accurately measured without interference.

There are two primary modes of measurement in an electrochemical cell: voltammetric and amperometric. Voltammetry scans a defined voltage range and measures the current generated in response. This is used for redox-active molecules, e.g., ferrocene (which is frequently used to characterize electrochemical sensors), which exhibit characteristic oxidation and reduction peaks in the resulting current as the voltage is swept. Additionally, some biomolecules, such as dopamine or uric acid, have innate redox activity and can be sensed directly by observing changes in the peak current. Voltammetry has a high dynamic range and is therefore useful in applications for sensing analytes whose concentration is highly variable. However, it is relatively slow and cannot be said to be truly continuous, and it requires additional data processing and is limited in the number of analytes it can measure without adding redox labels. For analytes that require an enzyme or probe molecule, amperometry is used, in which a constant potential is maintained and the current is measured over time, with the current changing as the analyte is oxidized/reduced or binds the probe on the working electrode. Amperometric sensors are generally favored in biosensing due to their simplicity and versatility.

The accuracy of these methods depends on the noise in the system, and as many biofluids have varying pH and salt concentrations, this can be problematic for electrochemical biosensors. However, miniaturization of electrodes can increase signal-to-noise ratios and increase sensitivity. Many nanoscale electrochemical biosensors have been developed in recent years to accomplish this [138,139], and some have reached detection limits below 1 pg/mL [140,141,142].

A third measurement modality in electrochemical sensing is electrochemical impedance spectroscopy (EIS). EIS uses an alternating current, the frequency of which is scanned across a range. This is useful because high-frequency measurements sample the electron transfer rate, whereas low-frequency measurements yield information about mass transfer. Therefore, changes in the resultant current will be more specific to the properties of the particular analytes under study.

More recently, transistor-based electrochemical sensors are gaining popularity. Organic electrochemical transistors (OECTs) rely on the physics of solid-state electronic devices to measure the current–voltage relationship of the device. As with the electronics industry, which began with metal-oxide semiconductor field-effect transistors (MOSFETs), organic FETs use a gate electrode to apply a voltage to a channel, made of a polymer, between source and drain electrodes. The whole system is immersed in an electrolyte fluid, and so the properties of the polymer channel dictate the I–V response of the device. OECTs’ use of polymers as the channel makes them highly versatile, as these polymers can be chemically modified in any number of ways to change their electronic properties, and further functionalized with probe molecules to detect specific analytes. Recently, they have been used as biosensors, able to observe individual binding events [143]. The high sensitivity and versatility of OECTs make them appealing for TC applications.

Electrochemical sensors have been widely acknowledged as useful in the field of biosensing, with good detection limits and versatility in detecting a range of analytes. Additionally, the electronics industry has developed many contact-based connectors for electronic wires, allowing for easy scaling of such an electronics-integrated chip with analysis equipment, providing options for rapid development of integrated TC systems.

### 4.4. Photonic Sensing

Integrated photonic sensing platforms are an appealing alternative to electrochemical sensing. They are sensitive, label-free, and scalable due to the adoption of fabrication processes from the semiconductor industry. Compared with electrochemical sensors, photonic sensors are also lighter and require less power, making them ideal for scalable biosensing applications. Photonic sensors have been widely used for diagnostics [144,145,146], and various modes have been commercialized.

Integrated photonics uses waveguides to control the movement of light through a chip. Waveguides propagate light within them due to total internal reflection, resulting from the refractive index contrast between the waveguide and the external environment. Waveguide size is generally on the order of the wavelength of light being used, and for most applications, this is in the visible-to-infrared range. Thus, waveguides used in photonic integrated circuits are in the range of microns to sub-microns. Additionally, they are patterned onto chips using standard lithography techniques from the semiconductor industry, in a layered fashion, making complex waveguide patterns fit within a small footprint. Thus, light can be manipulated through a chip and pass through various sensing elements in a highly multiplexed way.

Integrated photonic sensors also take advantage of a phenomenon called evanescence. Though light is generally confined within a waveguide, the amount of contrast between the waveguide and environmental refractive indices allows a variable amount of the electromagnetic power to “leak out” into the environment. The effective refractive index of the waveguide thus becomes a combination of that of the waveguide material and that of the environment. If the surface of the waveguide is functionalized with a probe, such as an antibody, the effective refractive index is increased to a new baseline. Then, when analytes begin binding to their respective probes, the change in refractive index of the waveguide can be measured.

There are several types of integrated photonic systems that can use this property of light to sense biomolecules. One way that photonic biosensors work is by relying on periodic architecture (Bragg grating [147] or photonic crystals [148]) to produce spectral features, whose wavelength depends on the effective refractive index of the waveguides. In contrast, interferometry-based sensors (Mach–Zehnder interferometer [149,150]) determine the difference in phase between a control and sensing waveguide by measuring the amount of destructive interference between them when an analyte is bound to the functionalized waveguide.

Microcavity resonant structures, such as ring resonators, are a class of devices of particular interest. Photons in resonators circulate through the sensing elements many times (on the order of 10 [4,5], proportionate to the sharpness of the spectral peaks, called quality factor, or “Q”) before they couple back into the bus waveguide, interacting with the sensing environment for an extended period, which results in a more reliable measurement of the waveguide refractive index. The innate sensitivity of resonance-based photonics devices outpaces the best electrochemical sensors, allowing the detection of single analytes [151] and, because of this, achieving bulk refractive index sensitivities of greater than 200 nm/RIU [152] (the spectral shift in response to a change in refractive index of 1). Though microspheres have been demonstrated to have very high sensitivities to changes in refractive index, they are difficult to fabricate at scale [153], and two-dimensional lithographically produced ring resonators have been explored as the most practical option for resonance-based sensing.

Photonic ring resonators have been explored extensively as sensitive diagnostics. Since their suggested utility as biosensors in the early 2000s [154], many groups have sought to capitalize on lithography-based production of photonic ring resonator biosensors, starting primarily in silicon due to its prevalence in the semiconductor industry. Silicon has a high refractive index (3.48 at 1550 nm), allowing for excellent confinement of light. Silicon’s evanescent field has been shown to extend approximately 63 nm [155]. The evanescent field length depends on the refractive index contrast between the waveguide and environment, so alternate materials have been used, such as silicon nitride in our group and others [156,157]. A longer evanescent tail means more sensitivity to the environment; however, since functional chemistry monolayers are 0.5–1 nm and antibodies are only 10–15 nm long, anything beyond 20–30 nm might only serve to increase background fluctuations resulting from bulk refractive index changes in the media. Thus, waveguide material and geometry, as well as the functionalization chemistry, can be optimized to increase sensitivity to protein binding.

Continual development of ring resonator biosensors has led to diagnostic tests for Q-fever [158], Ebola [159], cancer [160,161], and others. Additionally, their fabrication has become streamlined, and multiple companies have started commercializing them as rapid diagnostics, including Genalyte (San Diego, CA, USA), SiPhox (Burlington, MA, USA), and Phlotonics (Rochester, NY, USA).

However, the high innate sensitivity of ring resonators to changes in environmental index is hampered by several factors. Fabrication processes have worked to maximize sensitivity by reducing waveguide loss and optimizing etch processes to maximally expose sensing waveguides to their environment. Waveguide geometry can also be tailored to expose the guided mode to an increased functional surface area, such as in a slot waveguide [162]. Here, the mode is localized in the space between two adjacent waveguides (a slot), which consists entirely of the liquid carrier rather than the solid waveguide. This increases the sensitivity of the mode to binding, since the binding is occurring within the mode itself. This has led to bulk RI sensitivities near 300 nm/RIU [162] and protein sensing at concentrations in the single nM regime [163]. Additionally, functionalization chemistry plays a role [164], and optimization of functionalization chemistry and process parameters must be carefully executed. As mentioned earlier, labels can also be used to enhance the signal when low quantities of analyte are bound, and this has been done to detect sub-pg/mL quantities of proteins [129]. However, the label-free nature of photonics is a large part of their appeal, particularly with regard to living systems like TCs, and so the avoidance of labels is preferred.

There are non-photonic limitations as well. As mentioned earlier, antibody–antigen binding kinetics restrict the lower limit of detection, as below a certain concentration, no analyte will remain bound to an antibody-functionalized surface. Additionally, transport becomes a limiting factor in low-concentration conditions. If the volume of the sample is sufficiently large, it will be difficult for the analyte molecule to find the probe molecules on the sensors. This is a function of the diffusion characteristics of the molecule and the microfluidic parameters of the device and the sensor geometry [165]. Progress has been made in optimizing microfluidic designs, but the transport of analytes remains a problem in diagnostics.

Another important consideration for the use of photonics in TCs is the way they interface with analytical equipment. Progress has been made to use grating couplers in a vertically-coupled platform to allow for easy access to microfluidic diagnostic devices. The properties of grating couplers, i.e., vertical light coupling, introduce a switchable platform for high-throughput assessment of analyte binding in a tissue chip platform. This has been applied to a diagnostic platform by the Miller group [157] and is being adapted for tissue chips, with the goal of quickly cycling through the optical light source/read head to obtain high volumes of data from many devices. This still requires the use of lasers and detectors that are large and expensive. However, integrated diode lasers and on-chip spectrometers have been developed [166,167] that could be used to make single-chip systems with small footprints and low power requirements.

As mentioned above, the electronics industry has thoroughly developed many varieties of electrical connections, allowing users to plug in wires from devices to analysis instruments. At present, photonics research relies heavily on physical alignment of optical fibers to waveguides on chips. This makes analysis harder, as alignment may drift over time, resulting in loss of power through the chip and changes in intensity not due to analyte binding, confounding results in interferometric sensors (but not resonance-based sensors). Designing fixtures to maintain alignment throughout an experiment is possible, but long-term solutions are desirable. A photonic “plug” is highly sought in the field, and researchers are currently working on making this a reality [168,169,170]. Photonic resonator sensors thus represent a highly desirable modality for sensitive, label-free, and multiplex sensing that is scalable and has low power requirements, making them ideal candidates for monitoring biomolecules in real time in tissue chips.

### 4.5. Optoelectrical Components to Modify Cells

In addition to sensing cellular products, optical and electronic components of TC devices can be used to influence cell behavior. The ability to manipulate light and electricity opens opportunities to control cells that are electrically active or have light-sensing components. For example, neurons operate with a set membrane potential, and when this potential is disrupted by changes in ionic flow through their membranes, ion channels open, allowing rapid depolarization of the membrane and triggering the firing of an action potential down its axon. While ion channels are usually induced by the presence of specific ligands such as neurotransmitters, direct application of an electric field can trigger a neuron to fire. Thus, researchers have used small electrodes arranged in spatial patterns, called microelectrode arrays (MEAs), to control the firing of neurons on a two-dimensional surface. This has been done with simple electrode arrays with high spatial resolution due to the small size (<50 μm) of the electrodes [171,172]. Additionally, OECTs have been implicated as potential MEA electrodes [173].

**Figure 6:**
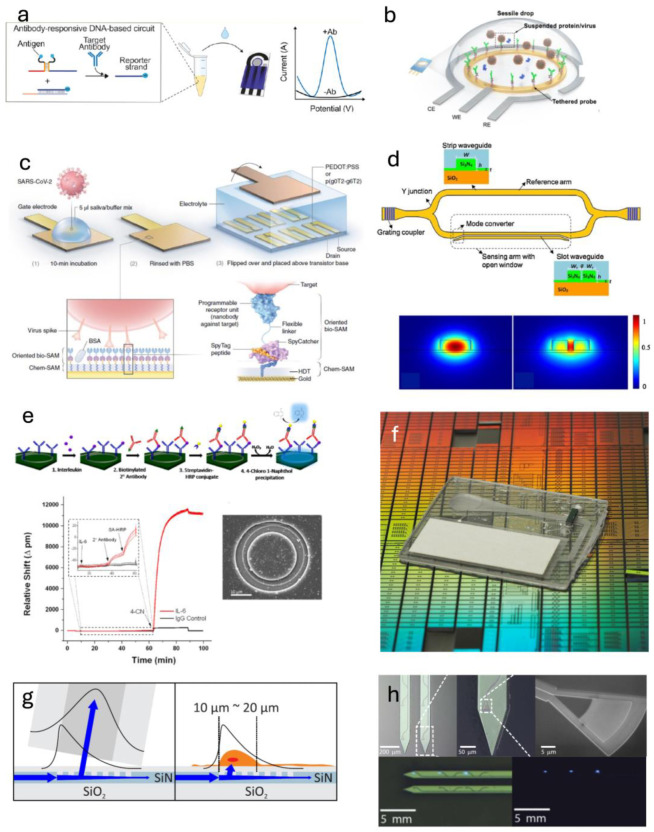
Examples of electrochemical and optical sensors that could be considered for TC integration. (**a**) An electrochemical sensor for DNA (Reprinted from Bracaglia 2021, Ref. [138] © American Chemical Society). (**b**) A sessile drop-based electrochemical sensor for viral particles (Reprinted from Zargartalebi 2022, Ref. [139] © American Chemical Society). (**c**) An OECT sensor that can detect single particles (Reprinted from Guo 2021, Ref. [143] © Springer Nature). (**d**) A slot waveguide Mach–Zehnder sensor (Reprinted from Liu 2013, Ref. [150] © Elsevier). (**e**) A ring resonator sensor using labels to achieve sub-picogram/mL detection limits (Reprinted from Kindt 2013, Ref. [129] © American Chemical Society). (**f**) Wafer-scale production of photonic ring resonator sensor chips for integration with passive microfluidic cards (Miller Group, 2022, unpublished). (**g**) Photonic grating couplers can be used to direct light to optogenetically-modified cells (Reprinted from Hoffman 2016, Ref. [177] © IEEE). (**h**) A probe containing photonic grating couplers for in vivo optogenetic stimulation (Reprinted from Shim 2016, Ref. [178] © Springer Nature).

In addition to the direct application of an electric field, light can be used to control neuron firing as well. By genetically modifying neurons to express light-sensitive membrane-bound proteins, called channelrhodopsins, light of a particular wavelength can trigger membrane depolarization. This concept is called optogenetics and has been exploited to trigger neurons in vitro [174,175] and to stimulate certain pathways in the brain in vivo [176]. Photonic grating couplers are periodic features in waveguides that direct light perpendicular to the waveguide and out of the chip, which has already been pursued [177,178] (Figure 6g,h). Photonic gratings tuned to the wavelength of the particular channelrhodopsins used in the cells and patterned across the surface of a photonic integrated circuit chip could serve the same purpose as an MEA, while using less power and with quicker responses.

While neural circuitry has obvious applications for optical and electrical stimulation with components of the TC device, many other cell types can be modified in this way. Cells have been shown to grow in response to voltages applied to gate electrodes of OECTs [179]. Spatially ordering cells in a device makes it possible to achieve the cellular architecture of complex organs, such as the layers of the cortex in the brain and its protruding white matter tracts, the layers of skin, pancreatic islets, or the ductile structures in the liver or salivary glands. Thus, active components of a TC are important for the development of complex biological models.

### 4.6. Toward an Integrated Biosensor Tissue Chip

**Figure 7:**
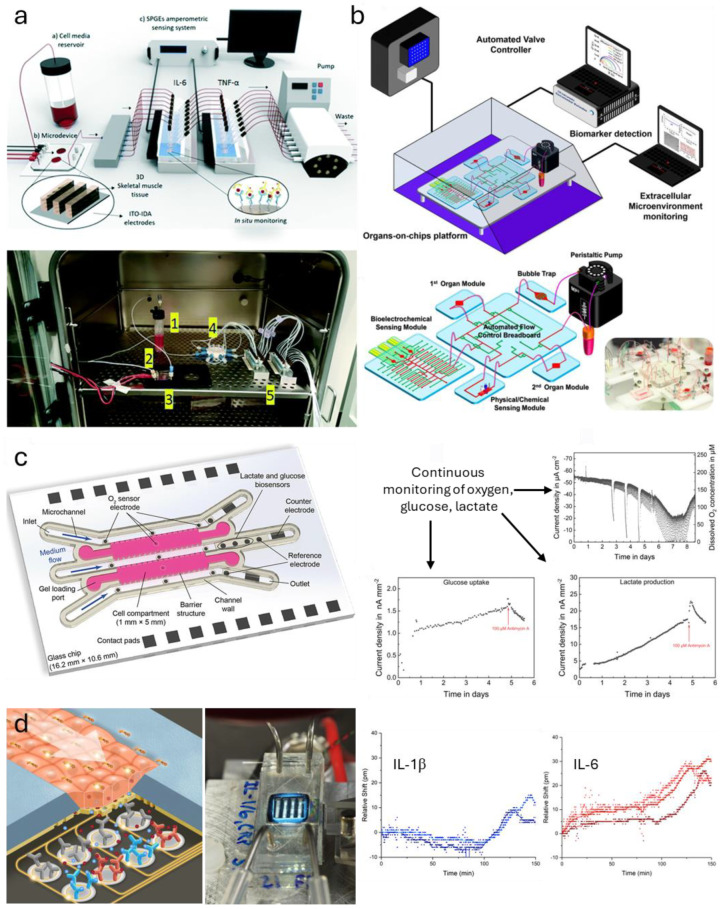
Sensor-integrated tissue chips. (**a**) In-line biosensors measure analytes from a muscle chip downstream from cells (Reprinted from Ortega 2019, Ref. [123] © Springer Nature). (**b**) A multi-organ chip measures analytes in a separate sensing module to determine drug efficacy and off-target effects (Reprinted from Zhang 2017, Ref. [39] © PNAS). (**c**) Electrochemical oxygen and metabolite sensors are incorporated directly into a microfluidic device, adjacent to channels containing gel-encapsulated breast cancer organoids. On the right, dissolved oxygen content, glucose consumption, and lactate production are monitored in real time (Reprinted from Dornhof 2022, Ref. [183] © Royal Society of Chemistry). (**d**) Sensors are incorporated directly below a tissue barrier for real-time sensing of secreted biomarkers. On the right, real-time data is shown for secreted IL-1B and IL-6 in response to LPS challenge (Reprinted from Cognetti 2023, Ref. [184] © Royal Society of Chemistry).

As mentioned earlier, some researchers have begun to incorporate protein-specific biosensors into TCs (such as Zhang et al. and Ortega et al., discussed earlier; shown in Figure 7a,b). A comprehensive list of such systems is shown in Table 1. However, they rely on downstream sensors separated from the cellular source of analytes by centimeters of tubing, resulting in significant dilution of analyte and again bringing strict sensitivity requirements into play. Additionally, this results in the loss of temporal resolution of secretion dynamics, as diffusion spreads the analyte throughout the available space before it reaches the sensor, and the sensor data becomes a combination of secretion dynamics, travel time through the fluidic architecture, and diffusion kinetics. Therefore, it is critical that future TCs begin to incorporate on-board biosensors, not as separate modules in serial microfluidic alignment with the tissue, but rather in immediate proximity, subject only to diffusion. Some groups have incorporated on-board protein [180,181] or metabolite [182] sensors into static models (e.g., in a well plate format), but this makes for less physiologically-relevant models and requires more manual manipulation, so microfluidic models are preferred.

The Weltin group has addressed these challenges by developing a microfluidic device with embedded electrochemical sensors for continuous real-time monitoring of oxygen and metabolites (glucose consumption and lactate production, using glucose and lactate oxidase-functionalized electrodes) [183]. Their use of multiparametric electrochemical sensors allows for the characterization of cancer organoids’ response to chemotherapeutic drugs in a clinically-relevant hypoxic cancer environment and represents a great advancement towards personalized medicine. Additionally, the use of enzymes for metabolite detection is highly versatile and can be modified for many analytes that have enzymes that act upon them in electrochemically measurable ways.

The Miller group recently demonstrated the feasibility of a similar approach applied to secreted proteins, using antibody-functionalized photonic ring resonators to sense cytokines secreted by bronchial epithelial cells in response to bacterial lipopolysaccharide challenge [184]. In this system, cells are cultured on a silicon nitride nanoporous membrane just 300 μm above a photonic ring resonator-based sensor chip. It was demonstrated via both finite-element modeling in COMSOL Multiphysics 6.0 and experimentally that, when the channel between the cells and sensor is not subject to flow, only a few minutes are required for secreted analytes to reach the sensor via diffusion. As expected, the close proximity of the sensor to secreted analytes from a monolayer of epithelial cells results in high concentrations relative to studies that sample media, in the hundreds of ng/mL to single μg/mL range. This simplifies the analytical performance constraints on the sensor, while providing better temporal resolution than a downstream or off-chip sensor. And as with metabolite enzymes, the use of antibodies makes this highly customizable to almost any protein analyte.

### 4.7. Challenges and Opportunities for Development

Many of the challenges in the sensor-integrated TC field are the same as in any sensor application. These include analytical performance of the sensor, manufacturing reproducibility, and shelf stability, among others. There are several additional issues that must be considered when integrating a sensor with a TC, however, and these are all research and development opportunities, with the potential to impact the field as it moves forward. First, capture probes, such as antibodies for sensors that rely on analyte capture, are typically chosen based on their high affinity and specificity for the analyte. As this inevitably means that the capture probe will have a very slow off-rate for its target, the potential for sensor saturation over the course of an experiment lasting days to weeks is quite high. To solve this challenge, methods for hot-swapping sensors or regenerating sensors in a manner that does not disturb the TC must be developed. One possibility is the use of switchable probes, as has been explored by the Soh group and others [185]. Second, the sensors we have discussed in this review primarily detect secreted analytes. To date, imaging methods using labeled probes are the only viable approach to monitor intracellular changes in a TC; are other approaches possible? Third, although most of the TCs described above use PDMS as the primary material of manufacture, increasing work in academia and industry focuses on manufacturing with laser-cut acrylic, injection molding, or 3D-printed microfluidic devices. Each material places specific constraints on sensor type, placement, and operation; all of these must be studied. Other issues associated with long-term sensor operation in a TC include biofouling as well as the increased potential for sensor drift. Both of these are issues that have been examined in the context of sensors for other purposes, but the potential need for long-term monitoring exacerbates the challenge. Finally, as one might expect, continuous sensing experiments running for days to weeks generate large datasets. Systems employing multiplex sensors and/or multimodal sensing create an even greater challenge in terms of data storage and analysis. Given the rapid rise of artificial intelligence (AI)-based solutions in other areas [186,187], including sensors [188], it is a certainty that AI-based systems for signal processing and analysis will find utility in sensor-integrated TCs.

## 5. Comparison and Conclusions

Careful consideration must be given to the needs of the TC system before choosing what sensor type to incorporate. For small molecule metabolites, redox-active enzymes are the preferred method of detection. Photonic sensors based on refractive index changes are mass-based and are thus best for binding reactions (i.e., antibody–antigen or nucleic acid hybridization) involving large molecules. Photonic chip-based spectroscopy methods (IR and Raman) may provide opportunities for small molecule sensing in TCs, but this has yet to be demonstrated. Photonic sensors tend to have an advantage in sensitivity, but as discussed in Section 4.1, this may not be important for on-chip sensors due to the proximity of the sensors to the cells and small microfluidic channel volumes. For TC applications, label-free operation is strongly preferred to avoid disruption to the flow of experiments or interference from label molecules, and both photonic and electrochemical sensors are label-free in their most basic form. However, they may be enhanced with labels if necessary. Both methods are susceptible to fouling and sensor drift, and controls for these issues must be carefully derived. Photonic sensors must be controlled for temperature, whereas electrochemical sensors may require shielding from electromagnetic interference. For TCs, consideration of materials is vital, since many materials are incompatible with most microfluidic device fabrication techniques. For example, Metrohm’s (Herisau, Switzerland) Dropsens electrode chips are challenging to integrate with microfluidics due to their ceramic coating. Additionally, chips built on silicon substrates will be opaque to visible light, making microscopy difficult. In this regard, electrochemical chips are advantageous as they can easily be deposited on glass or optically transparent plastic substrates. However, silicon-based chips may still be used by designing microfluidic devices that place sensors close to cells but offset from the visual path.

In academic settings, cost will be a major driver in determining which sensor type is best. Photonic sensor chips require a cleanroom environment and heavily optimized fabrication parameters, so collaboration with a foundry is preferred if not necessary. Electrochemical sensor chips can be produced in a similar manner, but their fabrication is less complicated. In some cases, screen printing is possible, greatly reducing cost but sacrificing performance and increasing variability between chips. Ultimately, the cost will come down to readout equipment. Electrochemical sensors require a potentiostat and connectors, which can be customized using pogo pins and many commercially available connectors. Photonic devices currently require a more costly setup, including a spectrometer, a tunable laser source, vibration isolation, temperature control, optomechanical breadboards and stages, micromanipulators, and possibly microscope lenses for visual alignment of fibers with waveguides.

Tissue chips represent an important use of microfluidic technology and cell-culture techniques to greatly enhance the quality and “human relevance” of knowledge gained from in vitro studies. Advanced imaging and incorporation of physical and chemical sensors have further validated the importance of in vitro work with tissue chips. In addition to simpler physical sensors, which have been incorporated into microfluidic cell-culture systems for years, researchers now have many tools at their disposal for sensitive detection of specific bioanalytes within a tissue chip. By carefully choosing appropriate probe molecules, microfluidic configurations, and sensor modalities and designs, the many available permutations can be used to tackle the many biological problems tissue chips are addressing, including the basic biochemistry of disease, new approaches to drug discovery, and understanding human development.

**Table 1:** Sensor- and actuator-integrated tissue chips. Notes: As analytical performance is generally not provided in the referenced articles, this is not included in the table. “Continuous?” indicates when measurements are taken continuously for the duration of the experiment. In some instances, sensors are able to function continuously, but the authors chose to take discrete measurements, so this was marked as not being continuous. “Multiplex Capability” refers primarily to the number of biological replicates on a chip. In instances where microfluidic circuits have multiple chips or individual sensor chips have multiplexing capabilities, this is described in parentheses.

Sensor Output Type	Tissue Type	Description	On-Chip?	Continuous?	Measurement Duration	Multiplex Capability	First and Corresponding Authors	Year	Ref.
*Chemical* *(pH, O_2_)*	Heart	Deposited iridium oxide thin film electrodes are used to measure the acidification of single cardiomyocytes in a valved microfluidic device.	Yes	Yes	∼2.5 h	Single	Ges, Baudenbacher	2008	[95]
	Adipose	Real-time (1 min. resolution) measurements of absorbance at two wavelengths, used to calculate pH from phenol red-containing media, using separate LED and sensor components positioned on either side of a transparent microfluidic device.	Yes	Yes	14 days	Single	Rajan, Lekkala	2016	[98]
	Bacterial culture	Chitosan hydrogel films swell in response to changes in pH; the thickness change is measured with optical reflectometry in 10 s intervals. This is used to track bacterial proliferation in suspended culture.	Yes	Yes	∼3.5 h	Single	Tang, Wu	2013	[81]
	Circulating tumor cells	Deposited zinc oxide electrodes are used to measure the pH of circulating cells of three different lines of circulating tumor cells (human lung, murine aorta, and canine kidney epithelium). Sensitivity of 48 mV/pH.	Yes	Yes	∼4 min	Single	Mani, Tsuchiya	2017	[96]
	Intestine	Intestinal epithelium in a two-channel microfluidic device with oxygen-impermeable coating to simulate partial hypoxia. Colorimetric nanoparticle coatings (with changes measured optically) measure oxygen concentration periodically (measurement takes 2 min., and device must be removed from flow setup).	Yes	No	24 h	Single (four sensors, two in each channel)	Grant, Ingber	2022	[80]
	Pancreas	Deposited platinum-based oxygen-sensitive dye is used to continuously monitor oxygen concentration and consumption in response to glucose stimulation, allowing inferral of metabolic activity.	Yes	Yes	∼4 h	Single (single measurement of multiple islets)	Schlünder, Loskill	2025	[87]
	Liver, kidney, artery	Multiple chips connected in parallel with oxygen control through incorporated scavenger modules and optical ruthenium dye-based microbead coatings as oxygen sensors, in a separate oxygen sensing module upstream of various specific organ modules. * Oxygen monitoring is continuous.	No	Yes *	7 days	Single (three separate oxygen sensing modules connected in parallel, with three different TCs in series)	Jiang, Zhang	2024	[78]
*TEER*	Lung epithelium	Epithelial barrier suspended between two microfluidic channels with integrated TEER electrodes. Impedance measurements were not continuous (every ∼5 min for disruption experiments; once per day for long-term experiments).	Yes	No	>60 days	Single	Henry, Ingber	2017	[83]
	Kidney epithelium (canine)	Cells cultured directly on a microelectrode array (MEA) with 20 µm resolution; different electrical parameters measure attachment, cell–cell adhesion, and metabolic activity. Individual measurements took 5 min, but were only taken at 24, 48, and 72 h.	Yes	No	3 days	Single	Abbott, Park	2022	[118]
	Various	High-throughput analysis of many tissue chips, with O_2_ and TEER sensors in addition to imaging. While TEER and O_2_ sensors are capable of real-time monitoring, measurements are actually taken every 5–30 min, dependent upon the use of multiple assays and allowing for equilibration with the environment, or once per day for long-term measurements. * TEER electrodes on chip, but in the ports far from the sample.	Yes *	No	Up to 11 days	96-plex (interfaced with 384-well plate)	Azizgolshani, Charest	2021	[42]
	Intestinal epithelium	Movable electrode for spatially-resolved TEER across a suspended epithelial barrier.	Yes	No	5 days	Single (four different locations in channel, measured in series)	Renous, Maoz	2021	[84]
	Intestine	Semi-high throughput model based on the Mimetas OrganoPlate^®^. TEER measurements were taken with the OrganoTEER^®^ module, also offered by Mimetas. Additionally, periodic cytokine assays were performed off-chip from sampled media (Luminex, Genk, Belgium). * TEER measurements on-chip, cytokine assays off-chip.	Yes *	No	11 days	40-plex	Beaurivage, Kurek	2019	[119]
	Cochlea	Semi-high throughput in a 96-well plate format. Gold electrodes are incorporated into both channels of the device for continuous TEER measurements. TEER was only measured at the beginning and end of the 14-day experiment.	Yes	No	14 days	16-plex	Bai, Shvartsman	2023	[116]
	Blood–brain barrier	A multiplex electrode array is used to continuously monitor TEER across a multichannel coculture BBB model in real time across the entire duration of the experiment.	Yes	Yes	5 days	16-plex	Jeong, Han	2018	[114]
	Blood–brain barrier	Multichannel BBB model that used chopstick-like electrodes to measure TEER values. Because of the high-throughput design, measurements were not continuous (4 h apart) because the electrodes had to be moved through the many ports. * For TEER, one electrode used the same port for each set of four endothelial cell modules, but the other electrode used unique ports for the corresponding four astrocyte modules.	Yes	No	∼3 days	64-plex *	Xu, Qin	2016	[115]
	Blood–brain barrier	Gold electrodes patterned on a polycarbonate porous membrane are used to perform electric cell–substrate impedance sensing on a layer of human IPSC-derived brain endothelial cells (and hIPSC-derived astrocytes on the other side of the membrane) in response to nitrosative stress. * ECIS is cyclic, with 2 min resolution.	Yes	Yes *	∼90 min	Single	Matthiesen, Herland	2021	[116]
Electrochemical	Liver, heart	Modular assembly of multiple organoid platforms (liver and heart), peristaltic pump, and bubble trap, with downstream physical/chemical (temp., pH, O_2_) sensing and biosensing (albumin, GST-a, CK-MB) modules, connected to a microfluidic breadboard via tubing. Sensors are capable of being regenerated upon saturation. Reported LODs of 2–90 pg/mL for the electrochemical protein sensors.	No	No	5 days	Single (two organ chips, plus multiplex sensor chips—single replicates of each of the three biomarkers)	Zhang, Khademhosseini	2016	[39]
	Muscle	Electrical (via MEA) and biological stimulation of muscle cells, and antibody-based electrochemical detection (with electroactive enzymatic sandwich detection antibody) of secreted protein analytes (IL-6 and TNF-α) in a separate downstream sensing module. LODs of 8 and 2 ng/mL range.	No	No	Up to 48 h	Single (one TC device, with multiplexed sensor: two analytes detected in series, with each analyte having eight replicate sensors)	Ortega, Ramón-Azcón	2019	[123]
	Breast cancer	Electrochemical sensors are adapted for continuous monitoring of dissolved oxygen, and enzymatic glucose/lactate detection for the monitoring of glucose consumption and lactate production in breast cancer organoids under hypoxic conditions and in response to doxorubicin treatment. LODs were 7.6 and 6.1 µM for glucose and lactate, respectively.	Yes	Yes	6 days	Single (integrated sensor chip has 10 replicates for oxygen, plus single-replicate glucose and lactate sensors)	Dornhof, Weltin	2022	[183]
Plasmonic/ photonic	Colorectal cancer organoid	Tumor organoids are arrayed on an antibody-functionalized plasmonic gold nanohole array. Secretion of VEGF-A is measured in real time under hypoxic conditions and chemotherapeutic drug treatment. LOD of 157 pg/mL. * Detection wells are separated from organoid laterally by 70 µm by a micropillar array since cells directly on top of the nanohole array will greatly affect the plasmonic signal.	Yes *	Yes	20 h	Up to ∼90-plex (100 microwells to encapsulate organoids, but some are kept empty and used as a reference)	Liu, Altug	2024	[181]
	Lung epithelium	Antibody-based photonic ring resonator sensors are immediately adjacent to the cell layer in the bottom channel of the microfluidic device. Multiplex detection of IL-1β, IL-6, and CRP with LODs of 3.1 and 7.6, and 1.5 ng/mL, respectively.	Yes	Yes	3 h	Single (one TC with sensing of two analytes simultaneously (three replicates)	Cognetti, Miller	2023	[184]
MEA/ Electro- chemical	Kidney epithelium (canine)	OECTs are used to form an electrochemical gradient along the length of a channel, with consequent localization of epithelial cells.	Yes	NA	NA	Single	Bolin, Berggren	2009	[179]
Optogenetic	Neuronal	Multichannel microfluidic device with integrated LED to stimulate optogenetically modified neurons; measured downstream oligodendrocyte maturation.	Yes	NA	Continuous stimulation pulses for 1 h daily for up to 14 days	Three-plex (where each culture setup contained two wells, illuminated by six total LEDs, one for each well)	Lee, Yang	2016	[174]
Combined optogenetic/ MEA	Neuronal, cardio- myocyte	Demonstrates medium-high throughput of dual LED stimulation and MEA electrical activity recording in static culture. MEA provided by Axion Biosystems (Atlanta, USA). * Individual recordings were continuous, but they were taken at least one day apart.	Yes	No *	12 days	48-plex (192 LEDs in 48 groups of four different wavelengths; MEA has 768 electrodes with 16 per well)	Clements, Ross	2016	[175]
